# The PPARβ/δAgonist GW0742 Relaxes Pulmonary Vessels and Limits Right Heart Hypertrophy in Rats with Hypoxia-Induced Pulmonary Hypertension

**DOI:** 10.1371/journal.pone.0009526

**Published:** 2010-03-04

**Authors:** Louise S. Harrington, Laura Moreno, Anna Reed, Stephen J. Wort, Béatrice Desvergne, Christopher Garland, Lan Zhao, Jane A. Mitchell

**Affiliations:** 1 Cardiothoracic Pharmacology, NHLI, Imperial College London, United Kingdom; 2 Critical Care Medicine, NHLI, Royal Brompton Hospital, London, United Kingdom; 3 Center for Integrative Genomics, University of Lausanne, Genopode, Lausanne, Switzerland; 4 Department of Pharmacology, University of Oxford, Oxford, United Kingdom; 5 Experimental Medicine and Toxicology, Hammersmith Hospital, Imperial College London, London, United Kingdom; Universidad Peruana Cayetano Heredia, Peru

## Abstract

**Background:**

Pulmonary vascular diseases are increasingly recognised as important clinical conditions. Pulmonary hypertension associated with a range of aetiologies is difficult to treat and associated with progressive morbidity and mortality. Current therapies for pulmonary hypertension include phosphodiesterase type 5 inhibitors, endothelin receptor antagonists, or prostacyclin mimetics. However, none of these provide a cure and the clinical benefits of these drugs individually decline over time. There is, therefore, an urgent need to identify new treatment strategies for pulmonary hypertension.

**Methodology/Principal Findings:**

Here we show that the PPARβ/δ agonist GW0742 induces vasorelaxation in systemic and pulmonary vessels. Using tissue from genetically modified mice, we show that the dilator effects of GW0742 are independent of the target receptor PPARβ/δ or cell surface prostacyclin (IP) receptors. In aortic tissue, vascular relaxant effects of GW0742 were not associated with increases in cGMP, cAMP or hyperpolarisation, but were attributed to inhibition of RhoA activity. In a rat model of hypoxia-induced pulmonary hypertension, daily oral dosing of animals with GW0742 (30 mg/kg) for 3 weeks significantly reduced the associated right heart hypertrophy and right ventricular systolic pressure. GW0742 had no effect on vascular remodelling induced by hypoxia in this model.

**Conclusions/Significance:**

These observations are the first to show a therapeutic benefit of ‘PPARβ/δ’ agonists in experimental pulmonary arterial hypertension and provide pre-clinical evidence to favour clinical trials in man.

## Introduction

Pulmonary hypertension comprises a spectrum of disorders with a multitude of aetiologies. The condition is defined clinically as a mean pulmonary artery pressure (mPAP) of greater than 25 mmHg (3.3 kPa) at rest [1]. Pulmonary hypertension is characterised pathologically by pulmonary arterial vasoconstriction, vascular remodelling and intraluminal thrombosis. These features predominantly affect small resistance pulmonary arterioles leading to a clinical picture of insidious dyspnoea progressing in parallel with diminishing pulmonary artery luminal diameter and increasing pulmonary vascular resistance. In the early stages the thin walled right ventricle is able to compensate by working harder leading to right ventricular hypertrophy. Eventually however, the adaptive capability of the right ventricle is exceeded with the development of right ventricular failure and subsequently death. Untreated, idiopathic pulmonary hypertension has a high mortality with a median survival of just 2.8 years and a 5 year survival rate of only 34% [2].

Intensive research efforts have focussed on the identification of aberrant pathophysiological signalling pathways at the level of the pulmonary arteriole. Vasoconstriction and the drive to remodel are limited by the release of vaso-protective hormones from the endothelium. These hormones include nitric oxide (NO) and prostacyclin. The endothelium also produces a powerful constrictor hormone, endothelin (ET)-1, which additionally stimulates smooth muscle cells to proliferate and vessels to remodel [3].

Pulmonary hypertension is associated with deficiencies in these pathways; an underproduction of dilator hormones and/or an overproduction of constrictors. In line with this, the current therapies available to treat pulmonary arterial hypertension are based on pharmacological intervention of each of these endothelium-derived hormones [4].

Prostacyclin and prostacyclin mimetics are a cornerstone of therapy for patients with pulmonary hypertension. They have been shown to improve exercise capacity and pulmonary haemodynamics, as well as showing long term survival advantage [5], [6]. An important downside of prostacyclin therapy is that it must be administered via continuous intravenous or subcutaneous infusion, or via multiple inhaled treatments throughout the day and night. This is not only inconvenient for patients, but interruption of an intravenous infusion may cause fatal rebound pulmonary arterial hypertension.

Prostacyclin formed by activated vessels [7], acts via cell surface IP receptors linked to activation of adenylate cyclase. Recent evidence suggests that prostacyclin could also be a ligand for the nuclear PPARβ/δ receptors which act to modulate gene expression [8], [9]. In addition, we have recently shown that the prostacyclin mimetic treprostinil sodium, which is currently licensed for the treatment of pulmonary hypertension, activates PPARβ/δ receptors in lung fibroblasts [10] and in human platelets [11]. There are three PPAR receptors; PPARα, PPARβ/δ and PPARγ. Orally active PPARα and PPARγ agonists are already used in clinical practice for the treatment of hyperlipidaemia and type 2 diabetes. They are well tolerated and have a good safety profile. Moreover, pre-clinical studies have shown that the PPARγ ligands rosiglitazone, pioglitazone and troglitazone have some protective effects in the chronic hypoxia and monocrotaline models of pulmonary arterial hypertension in rats [12], [13], [14] with significant reductions in pulmonary vascular remodelling in both these models. However, the possibility that PPARβ/δ agonist may affect pulmonary hypertension has not yet been addressed.

Thus, here we investigated the effects of PPARβ/δ agonists (including GW0742) on pulmonary artery tone in vessels from rats and mice. We have compared responses in pulmonary arteries with those seen in mesenteric arteries and the aorta. We have also used vessels from genetically modified mice where IP or PPARβ/δ genes have been deleted to address the role of each receptor in responses induced by PPARβ/δ agonists. The effect of PPARβ/δ agonists on cAMP, cGMP, membrane potential or Rho kinase activity in arterial vascular tissue was studied. Finally, we investigated the effects of the PPARβ/δ agonist GW0742 on markers of pulmonary hypertension induced by hypoxia in rats.

## Methods

### Myography

Male C57BL/6 mice (20–30 g) were killed by lethal exposure to carbon dioxide followed by cervical dislocation. The mice were maintained and killed in accordance with Home Office guidelines for the use of experimental animals. The heart and lungs were removed as a block and placed into physiological salt solution (PSS; composition in mM) containing NaCl 119, KCl 4.7, CaCl_2_ 2.5, MgSO_4_ 1.17, NaHCO_3_ 25, KH_2_PO_4_ 1.18, EDTA 0.027 and glucose 5.5. The heart lung block was pinned out in a dissecting dish containing PSS, to allow the descending thoracic aorta to be carefully cleaned of fat and connective tissue. Following this, the first order pulmonary arteries were identified and carefully dissected from surrounding structures, connective tissue and fat. These arteries were stored in fresh PSS at room temperature until use. The mesenteric bed was removed, pinned out on a silicon based Petri dish before being cleaned of fat and connective tissue. Second order arteries were dissected and loaded on to wire myographs as described previously [15]. For some experiments 3^rd^/4^th^ order pulmonary artery from male Wistar rats (250–270 g) were used. For other studies, responses of vessels from genetically modified male mice in which either the IP or PPARβ/δ receptors were deleted (IP^−/−^ or PPARβ/δ^−/−^) were compared with those seen in vessels from wild type control animals. IP^−/−^ mice were a generous gift from Professor Maria Belvisi and Dr Sarah Maher located locally at Imperial College [16]. Colonies of PPARβ/δ^−/−^ mice were generated by Professor Béatrice Desvergne [17]. Vascular tissue from PPARβ/δ ^−/−^ and the corresponding wild type mice were collected from Switzerland and transported at room temperature in sterile DMEM (0% FCS) before being used (within 6 hours) experimentally in London. For myography experiments n-values refer to tissues from individual animals.

### Isometric Myograph Recordings

Using 2 tungsten wires (diameter 40 µm), 2 mm long segments of artery were mounted in a four channel Mulvany-Halpern myograph (Model 610 M, Danish Myo Technology, Aarhus, Denmark) and supported in PSS, at a temperature of 37°C, bubbled with 95% O_2_ and 5% CO_2_. After 30 minutes the tension of the aortic segments and mesenteric arteries were normalised to a tension equivalent to that generated at 90% of the diameter of the vessel at 100 mmHg, using standard procedures as described previously [15]. The pulmonary artery segments were normalised to a tension of 7.5 kPa which is roughly half that of the aortic segments given that the pulmonary circulation is exposed to lower pressures *in vivo*. Changes in arterial tone were recorded via a PowerLab/800 recording unit (ADinstruments). In order to assess the viability of vessels they were first challenged twice with high potassium solution (KPSS; composition in mM: KCl 123.7, CaCl_2_ 2.5, MgSO_4_ 1.17, NaHCO_3_ 25, KH_2_PO_4_ 1.18, EDTA 0.027 and glucose 5.5) which induces vasoconstriction in a viable tissue.

### Drug Treatments

#### Tissues contracted with U46619 or with phenylephrine

Cumulative contractile concentration response curves to the thromboxane mimetic U46619 were constructed (1 nM to 1 µM) before the myograph baths were washed out three times with PSS and the blood vessels allowed to equilibrate back to baseline. Vessels were then precontracted with the EC_80_ concentration of U46619. Once a stable contraction was achieved, cumulative concentration response curves to the PPARβ/δ agonist GW0742 (1–100 µM), and other specified vasodilators were constructed in pulmonary artery, mesenteric artery and aortic vascular rings. The vehicle control for GW0742 was DMSO with the final concentration in experiments using mouse tissue being 0.6% v/v and in experiments using rat tissue being 0.8% v/v, where vessels were contracted with U46619, or 0.4% where contracted with hypoxia. In some experiments the contractile agent phenylephrine was used in place of U46619. As with U46619, where phenylephrine was used, cumulative contractile concentration response curves (1 nM to 10 µM) were constructed to determine the EC_80_ concentration for each tissue. In additional experiments, selective inhibitors were added between 15–45 minutes prior to addition of the contractile agent, and dilator experiments repeated as above.

#### Tissues contracted with hypoxic

*In vitro* hypoxia elicits a transient constriction of pulmonary arteries within 1–2 min. This is followed by a more sustained response which, in our setup, decays over 40 minutes and, as such, protocols using hypoxia as a contractile stimuli, are time limited. For this reason, just two concentrations of GW0742 were tested as relaxants of hypoxia-induced vasoconstriction. For these experiments 3^rd^/4^th^ order rat pulmonary artery was used and mounted in myographs. Tissues were stretched to give an equivalent transmural pressure of 4 kPa as previously described [18], [19]. Vessels were then primed with submaximal concentrations of pheylephrine (30 nM) and further contracted with two successive challenges of hypoxia. The relaxant effects of GW0742 (10–30 µM) or vehicle (DMSO; 0.4%) were examined during the second, and more stable, exposure to hypoxia. For comparison, the relaxant effects of GW0742 on parallel pulmonary artery rings contracted with U46619 (100 nM) were studied.

#### Measurement of membrane potential

Smooth muscle cells of whole mesenteric arteries were impaled with sharp glass electrodes filled with 2 M KCl, tip resistances ∼80–100 MΩ, and membrane potential and tension were measured simultaneously [20]. Second-order rat mesenteric arteries were incubated with or without 100 mM L-N^G^-nitro-L-arginine methyl ester (L-NAME) for 20 min and precontracted with the EC_80_ concentration of phenylephrine. Once a stable contraction was obtained arteries were exposed to cumulative concentrations of GW0742, as above, and the tension and membrane potential were measured continuously for up to 20 min. At the end of the experiment 30 µM of acetylcholine was added as a control agent to induce membrane potential changes [20].

#### Measurement of adenosine 3′,5′ cyclic monophosphate (cAMP), guanosine 3′,5′ cyclic monophosphate (cGMP) or RhoA kinase activity in aortic rings

Aorta was removed and cleared of connective tissue as described above. Aortic rings of 2 mm width were cut and placed into individual wells of a 96-well culture plate containing 100 µl of Dulbecco's modified eagle medium containing 10% heat-inactivated fetal bovine serum, penicillin (100 U/ml), streptomycin (100 µg/ml), and non-essential amino acids (1% vol/vol) and allowed to equilibrate for 30 min in an atmosphere of 5% CO_2_ at 37°C and then, replaced with fresh medium. For these experiments n values were defined as separate segments of aorta, where for each experiment, tissue from at least 4 separate animals was used.

#### cAMP and cGMP assay

For measurement of cyclic nucleotides, the pan-phosphodiesterase inhibitor, 3-isobutyl-1-methylxanthine (IBMX) was then added in a volume of 10 µl to give a final concentration in the well of 300 µM for 15 minutes. Drugs or DMSO (final concentration of 0.03%) were then added in volumes of 10 µl to give the following final concentrations in the well: 10 µM sodium nitroprusside (SNP); 10 µM forskolin; 30 µM GW0742. Tissues were incubated for a further 20 minutes before the reaction was stopped and tissues lysed by replacing the medium with ice cold HCl (0.1 M). Protein content of extracted samples was measured with Bradford's reagent. Samples were then diluted 1∶3 with the assay buffer provided with the cAMP or cGMP ELISA kits (see below) to provide a final concentration in the range of 1-2 µg per well, depending upon individual experiments. cAMP and cGMP were measured using commercial enzyme-linked immunosorbent assay (ELISA) kits according to manufacturer's instructions (Cayman Chemicals, Ann Arbor, MI; cAMP and cGMP ELISA kits 581001 and 581021 respectively).

### RhoA Activation Assay

RhoA activation was measured using a commercial kit (G-LISA™ RhoA Activation Assay Biochem Kit™; from Cytoskeleton) which detects active GTP-bound RhoA. For these measurements aortic rings were placed in warm DMEM and allowed to equilibrate for 30 min, as above, followed by the addition (in 10 µl volumes) of Rho associated protein kinase (ROCK) inhibitor Y27632 or GW0742 at final concentrations in the well of 30 µM or vehicle (DMSO) for 20 min, before adding U46619 or vehicle in a volume of 10 µl to produce a final concentration of 10 nM and incubating for a further 15 min. The reaction was stopped by adding cold PBS (200 µl) and the aortic rings were snap–frozen using liquid nitrogen. Samples were homogenized using an Ultraturax homogenizer in lysis buffer containing the provided protease inhibitors. The homogenate was centrifuged at 10 000 *g* for 2 minutes at 4°C to remove cellular debris. Protein concentration was measured using the assay provided with the kit (Precision Red™ Advanced Protein) and 1.5 µg of tissue extract added per well. Data were expressed as fold change versus control (vehicle-treated) samples.

#### Ethics Statement

All animal procedures were carried out under specific protocols approved by the Home Office.

#### Chronic hypoxia model of pulmonary hypertension *in vivo*

Male Sprague-Dawley rats (Harlan, Indianapolis, IN) weighing between 230–250 g were randomised to normoxia (21% inspired O_2_; n = 10) or hypoxia (10% inspired O_2_; n = 29) for 21 days. They were treated with vehicle alone or vehicle plus the PPARβ/δ agonist GW0742 at 30 mg per kg for 23 days. This dose of drug was recommended by the supplier, GSK and consistent with other studies using the compound *in vivo* [21]. Fresh food, water and clean cages with bedding were provided twice weekly. Light was maintained on a 12 hour cycle and humidity was 55% with a temperature of 19–23 degrees Celsius. The animals were monitored daily and weight was checked. Vehicle or vehicle plus drug was administered once daily by oral gavage.

#### Haemodynamic measurements and tissue procurement

At the end of 3 weeks, rats were anaesthetised with fentanyl-fluanisone-midazolam (2.7 ml/kg body weight intraperitoneally). The ventral neck area was cleaned with 70% ethanol and an L shaped incision measuring 2 cm by 2 cm was made starting in the midline under the chin and extending to the distal aspect of the right clavicle in order to expose the right internal jugular (RIJ) vein. A precurved catheter was inserted into the RIJ and a pressure tracing was transduced and recorded by PowerLab Data Acquisition system (ADInstruments Limited, Oxfordshire, UK). To measure right ventricular systolic pressure the catheter was passed through the right atrium into the right ventricle. It should be noted that that whilst right ventricular systolic pressure is used as a surrogate for pulmonary artery pressure [22], [23], it is not a direct measure. A second catheter was inserted into the left carotid artery. This was located by blunt dissection just lateral to the midline position. The carotid systolic arterial pressure was recorded using the PowerLab Data Acquisition system. Following removal of the carotid catheter, fresh arterial blood was collected for measurement of haematocrit. The abdominal cavity was opened and rats were killed by exsanguination via the abdominal inferior vena cava. Withdrawn blood was centrifuged at 6000 rpm for 6 minutes to provide platelet free plasma. The chest was opened and left atrium removed. A needle was inserted into the right ventricle and 20 mls of heparinised saline (1∶100) were flushed through the lungs to clear the pulmonary vasculature of blood. The heart lung block was then removed from the chest. The right ventricle was carefully dissected from the septum plus left ventricle and each part weighed individually and used to calculate the ratio of wet right ventricle weight to wet septum plus left ventricle weight and body weight. The left lung was removed in its entirety and weighed. 10% formalin was injected into the bronchial tree via the left main bronchus and the inflated left lung was stored in formalin for later histological analysis.

#### Microscopy and lung morphometric analysis

Transverse sections of fixed lung were stained with Van Giessen and alpha actin stains to identify elastin and smooth muscle, respectively, in vessel walls. Distal pulmonary vessels (<100 µm) were counted and the degree of muscularisation for each vessel was assessed [24] as fully muscularised (two distinctive and continuous elastic lamina), partially muscularised (second elastic membrane not continuous <75%) and non—muscularised (single elastic membrane).

#### Materials

GW0742 was a kind gift from Timothy Willson, GlaxoSmithKline (USA). GW0742 was suspended in gum tragacanth (Sigma, UK) for administration via oral gavage, and diluted in DMSO for myography experiments. All other reagents were from Sigma (UK), unless otherwise stated.

## Results

### In Vitro Study of GW0742 in Blood Vessels

#### Relative responses of PPAR agonists as dilator agents in pulmonary artery, aorta and mesenteric vessels from rodents

**Figure 1:**
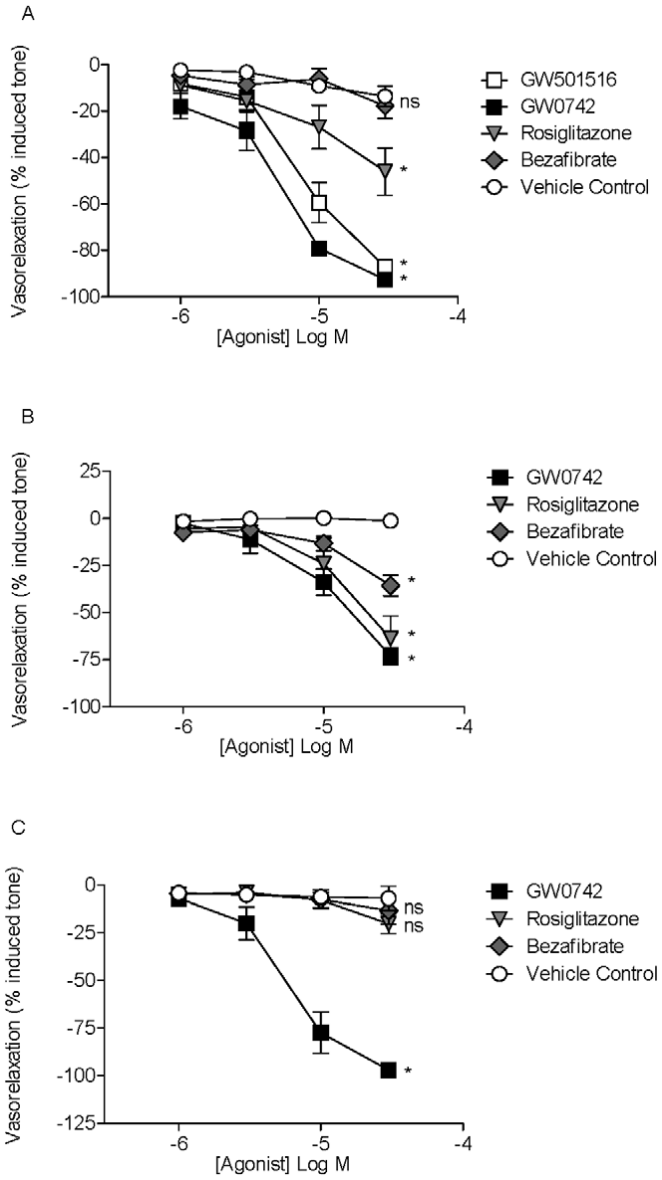
Effects of PPAR agonists on vascular tissue. The vaso-relaxant responses of three different PPAR agonists in (A) pulmonary artery, (B) aorta and (C) mesenteric artery. GW501516 or GW0742, which activate PPARβ/δ, rosiglitazone, which activates PPARγ or bezafibrate, which activates PPARα, were added to U46619 [EC_80_] contracted arteries in increasing concentrations. Responses of vehicle (DMSO; final accumulated maximum of 0.6%) are shown as ‘vehicle control’. The data is the mean ± standard error of the mean for n = 4−8 experiments. Drug induced responses were compared with time control using two-way ANOVA and a p value of <0.05 was assumed statistically significant and denoted by*. Where p>0.05, the lack of statistical significance is denoted by ns.

**Figure 2:**
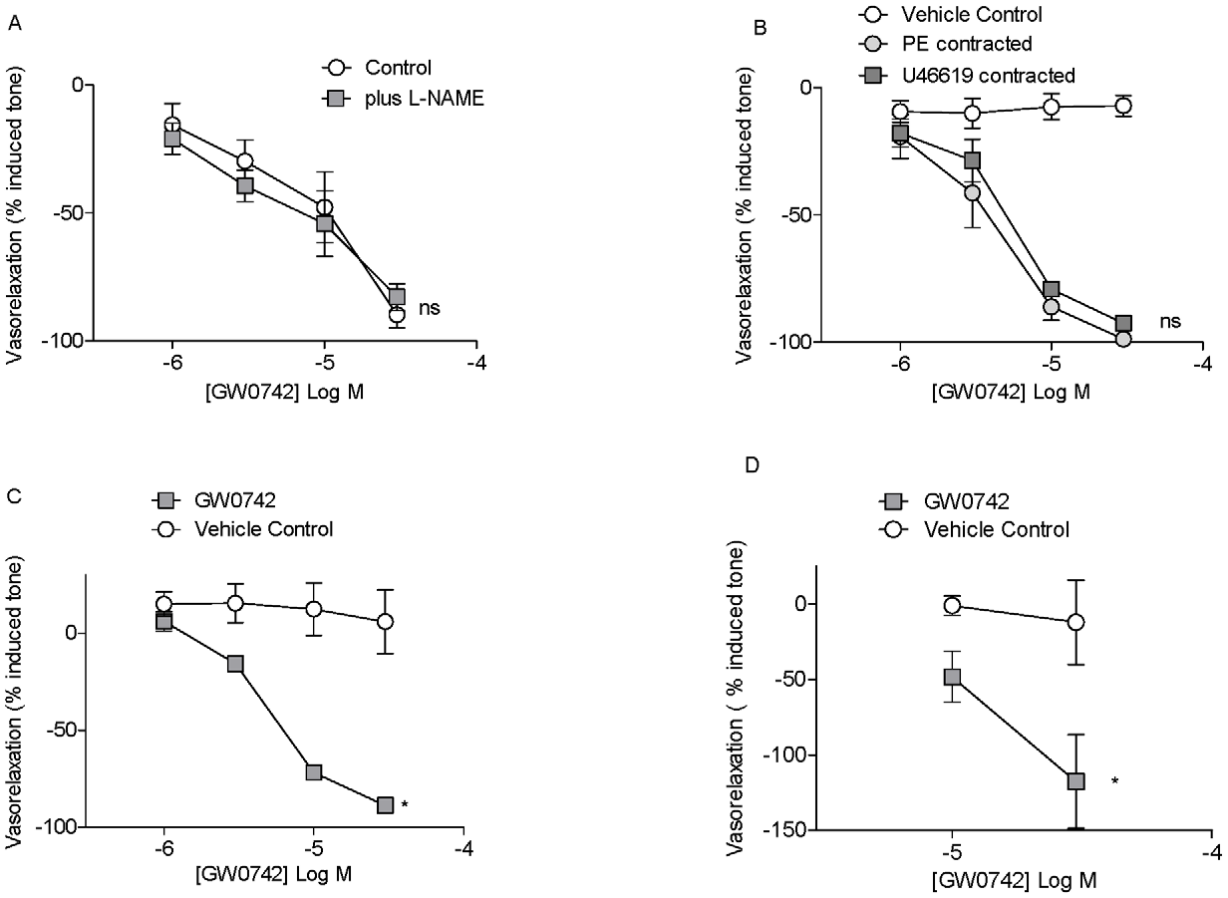
Characterisation of relaxant effects of GW0742 in pulmonary vessels. L-NAME (1 mM) had no effect on relaxant responses induced by GW0742 in U46619 contracted mouse pulmonary artery (A). GW0742 induced similar relaxant responses in mouse pulmonary artery contracted with EC_80_ concentrations of U46619 or phenylephrine (PE) (B). GW0742 induced relaxant responses in rat 3^rd^/4^th^ order pulmonary artery contracted with 100 nM U46619 (C) or with hypoxia (D), vehicle control for these experiments was again DMSO at accumulated maximum concentrations of 0.8 and 0.4% v/v respectively. Data is the mean ± standard error of the mean for n = 4−8. Drug induced responses were compared with time control using two-way ANOVA and a p value of <0.05 was assumed statistically significant and denoted by*. Where p>0.05, the lack of statistical significance is denoted by ns.

In U46619 contracted mouse pulmonary artery two chemically distinct PPARβ/δ agonists, GW501516 and GW0742, induced acute vascular relaxant responses (Figure 1a). GW0742 also induced relaxant responses in isolated pulmonary arteries from rats (Figure 2 c/d). The PPARγ agonist, rosiglitazone, also induced relaxant responses in mouse pulmonary artery, although its activity was less potent and efficacious than that seen with PPARβ/δ agonists (Figure 1a). In contrast to GW0742, GW501516 or rosiglitazone, the PPARα agonist bezafibrate did not induce significant relaxant responses in mouse pulmonary artery (Figure 1a).

Relaxant responses of the PPAR agonists were also examined using murine aorta (Figure 1b) and mesenteric artery (Figure 1c) vascular rings. In aorta, the PPARβ/δ agonist, GW0742, was an active vaso-relaxant, but was less potent and efficacious than in pulmonary artery. Moreover, in aortic tissue GW0742 was a similarly potent vaso-relaxant as rosiglitazone (Figure 1b). In the aorta bezafibrate produced small, but significant, relaxation responses. In mouse mesenteric arteries (Figure 1c) GW0742 induced potent and profound relaxant responses, similar to those seen in the pulmonary artery (Figure 1a). However, in the mesenteric artery neither rosiglitazone nor bezafibrate induced significant relaxant responses (Figure 1c).

#### Characterisation of vascular relaxation induced by GW0742

The dilator effects of GW0742 on mouse pulmonary artery were found to be independent of endothelial derived nitric oxide because pre-incubation of tissues with the nitric oxide synthase inhibitor L-NAME (1 mM) did not prevent responses (Figure 2a). Similarly in mesenteric artery and in aorta, removal of endothelium did not affect the relaxation induced by GW0742 (data not shown). In line with observations made using U46619, GW0742 induced similar relaxant responses when the pulmonary artery was contracted with phenylephrine (figure 2b). Similarly to results seen with mouse pulmonary artery, GW0742 relaxed 3^rd^/4^th^ order branch pulmonary artery from rats contracted with U46619 (Figure 2c) or with hypoxic challenge (Figure 2d).

#### Roles of prostacyclin IP and PPARβ/δ receptors in the vascular relaxation induced by GW0742

**Figure 3:**
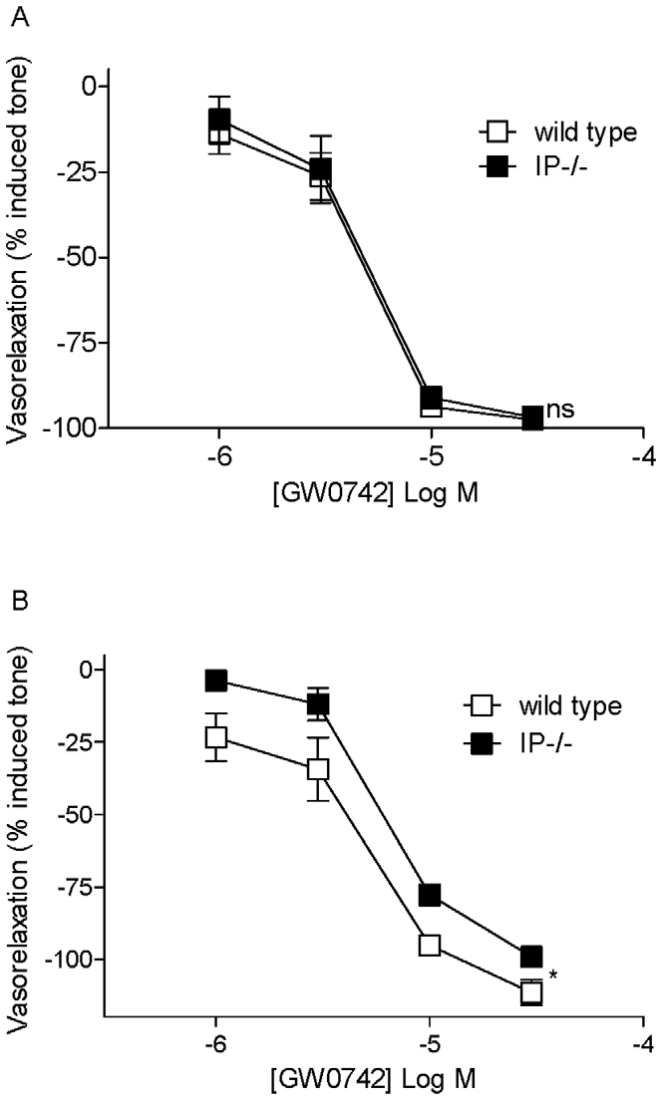
Role of IP receptors in the responses of PPARβ/δ agonists in blood vessels. Relaxations induced by the PPAR β/δ agonist GW0742 in (A) mesenteric artery and (B) pulmonary artery from wild type mice were compared to responses in tissues from IP^−/−^ mice. Vessels were pre-contracted with EC_80_ concentrations of U46618. The data is the mean ± standard error of the mean for n = 4−5 experiments. Drug induced responses were compared using two-way ANOVA and a p value of <0.05 was assumed statistically significant and denoted by*. Where p>0.05, the lack of statistical significance is denoted by ns.

**Figure 4:**
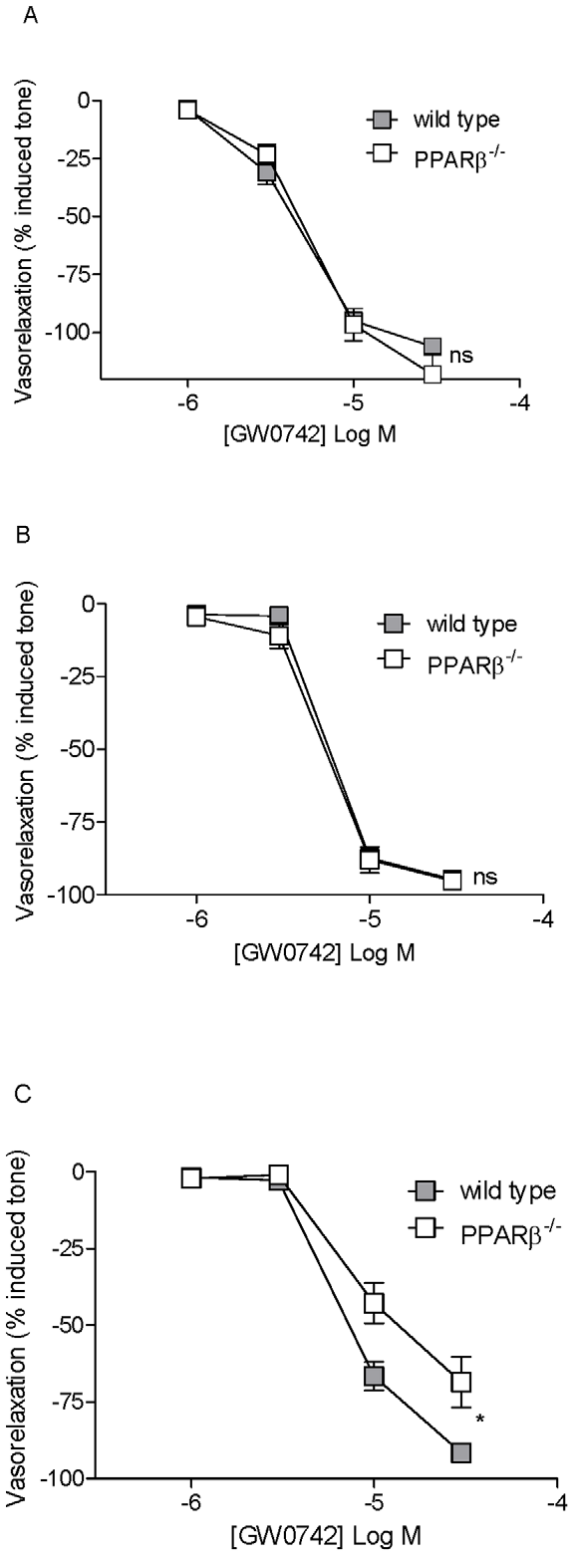
Role of PPAR β/δ receptors in the effects of GW0742 in blood vessels. Relaxations induced by the PPAR β/δ agonist GW0742 in (A) pulmonary artery, (B) mesenteric artery and (C) aorta from wild type mice were compared to tissue from PPAR β/δ^−/−^ mice. Vessels were pre-contracted with EC_80_ concentrations of U46619. The data is the mean ± standard error of the mean for n = 4 experiments. Drug induced responses were compared using two-way ANOVA and a p value of <0.05 was assumed statistically significant and denoted by*. Where p>0.05, the lack of statistical significance is denoted by ns.

The role of known prostacyclin receptors (cell surface IP and cytosolic PPAR β/δ receptors) in the relaxant responses induced by GW0742 was assessed by comparing responses in tissues from wild type and gene deleted mice. GW0742 induced relaxant responses in mouse pulmonary artery and mesenteric arteries from wild type and IP^−/−^ mice (Figure 3). The relaxation induced by GW0742 in mesenteric artery was unaffected by IP gene deletion (Figure 3a). The response induced by GW0742 in pulmonary artery from IP^−/−^ mice showed a small but significant reduction compared to responses in tissue from paired wild type mice (Figure 3b). Aorta was not studied from IP^−/−^ mice as, in this vessel, IP receptors are not linked to vaso-relaxation. As expected, and by way of a phenotype control, the relaxant response of treprostinil sodium (which activates PPAR β/δ receptors in some tissues and IP receptors in all tissues) was greatly reduced in mesenteric arteries from IP^−/−^ mice (data not shown). The relaxant effect of GW0742 on pulmonary artery (Figure 4a) and mesenteric artery (4b) was unaffected by deletion of the PPAR β/δ gene. However, by contrast, the relaxant effects of GW0742 in aorta was significantly blunted in tissue from PPAR β/δ^−/−^ mice compared to those in matched wild type control animals (Figure 4c).

#### Role of known smooth muscle relaxant pathways in the responses induced by GW0742 in blood vessels

**Figure 5:**
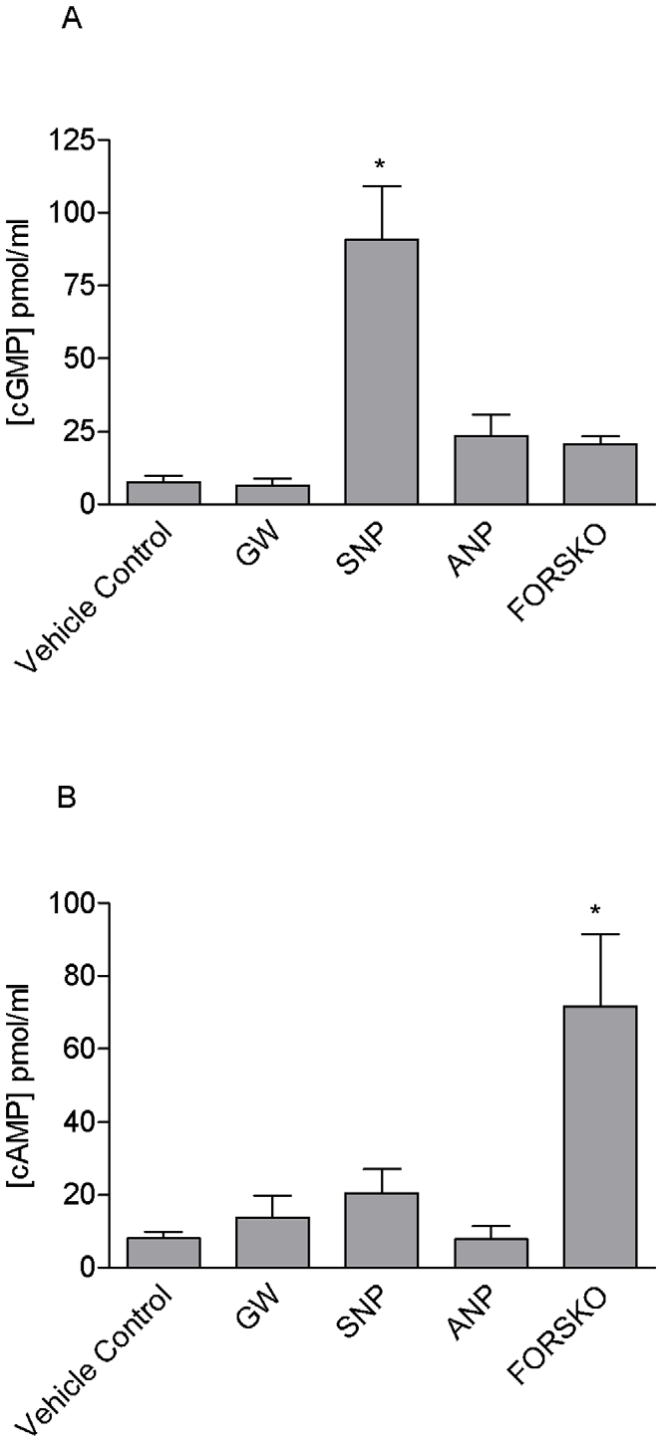
Effect of GW0742 on cGMP and cAMP levels in aortic rings. Effects of 30 µM GW0742 (GW), 10 µM sodium nitroprusside (SNP; an activator of soluble guanylate cyclase) and 10 µM forskolin (FORSKO; an activator of adenylate cyclase) on (A) cGMP and (B) cAMP levels in mouse aortic rings. The data is the mean ± standard error of the mean for n experiments. cGMP: vehicle control (DMSO; 0.03%) and GW0742, n = 14; SNP, n = 9; forskolin, n = 4. cAMP: DMSO and GW0742, n = 11; SNP, n = 6; forskolin, n = 7. Drug induced responses were compared using one-way ANOVA followed by Dunnett's Multiple Comparison Test. Statistical significance was assumed where p<0.05 and is denoted by *.

**Figure 6:**
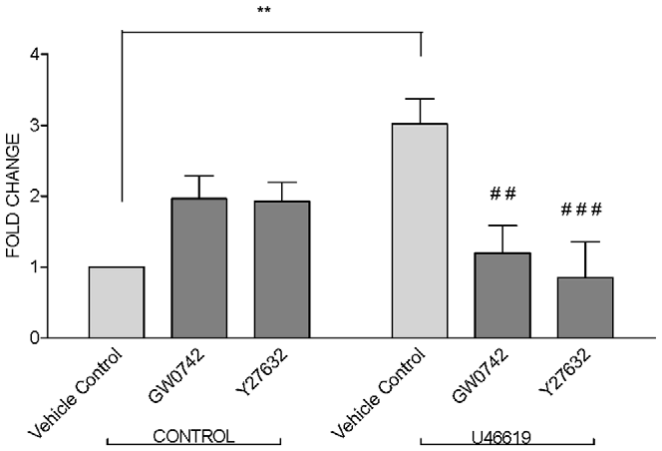
Effects of GW0742 on activation of Rho A in aortic rings. Mouse aortic rings were treated with or without U46619 (10 nM) before the additions of GW0742 and Y27632 (10 µM) or vehicle control (DMSO; 0.03%) and the content of GTP bound RhoA was measured in tissue extracts by ELISA. Data is the mean ± standard error of the mean for n = 4 experiments. Data was analysed using one sample T-test for normalized data or by one-way ANOVA followed by Bonferroni's Multiple Comparison Test. Significance was defined by **p<0.01 versus control and ##, ### p<0.01 and p<0.001 treatments with U46610 compared to respective control.

**Figure 7:**
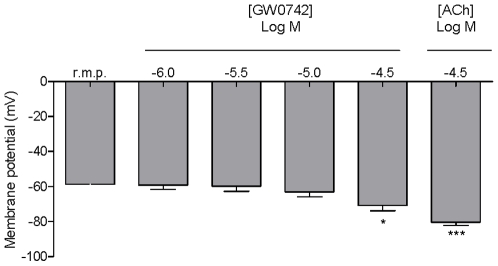
Effects of GW0742 on membrane potential in mesenteric arteries. Membrane potential in mesenteric arteries was measured under control conditions (resting membrane potential; r.m.p.) before GW0742 was added in a cumulative manner. Data is the mean ± standard error of the mean for n = 4. Data was analysed using one-way ANOVA followed by Dunnett's Multiple Comparison Test. Significance was assumed and defined by *p<0.05 and ***p<0.001 respectively.

Relaxation of blood vessels is generally mediated by at least one of four classic pathways. These include increases in cGMP or cAMP, activation/inhibition of ion channels resulting in hyperpolarisation of smooth muscle, or inhibition of RhoA. Vascular rings were prepared and incubated with GW0742 or activators/inhibitors of guanylate cyclase, adenylate cyclase or ROCK before reactions were stopped and samples extracted from the tissue. GW0742 did not cause any increases in cGMP (Figure 5a) or cAMP (Figure 5b). As expected, the nitrovasodilator sodium nitroprusside (SNP) increased cGMP (Figure 5a) levels whilst, forskolin, which activates adenylate cyclase, increased cAMP (Figure 5b). By contrast to observations with cGMP and cAMP we found evidence to suggest that GW0742 inhibits RhoA activity in vascular tissue. GTP-bound RhoA was increased in vascular tissue when stimulated with the known activator of this system, U46619. GTP-bound RhoA was inhibited by the classical inhibitor Y27632 and by GW0742 (Figure 6). In separate experiments we investigated the ability of GW0742 to induce hyperpolarization in segments of mesenteric artery by measuring membrane potential in the smooth muscle component of the tissue. GW0742 had no effect on membrane potential at concentrations up to 10 µM (Figure 7; which dilate this tissue by approximately 75%). However, at concentrations of 30 µM significant hyperpolarisation was noted (Figure 7). Maximum hyperpolarisation induced by acetylcholine (30 µM) is shown for comparison (Figure 7).

### In Vivo Study of GW0742 in a Rat Model of Hypoxia-Induced Pulmonary Hypertension

**Figure 8:**
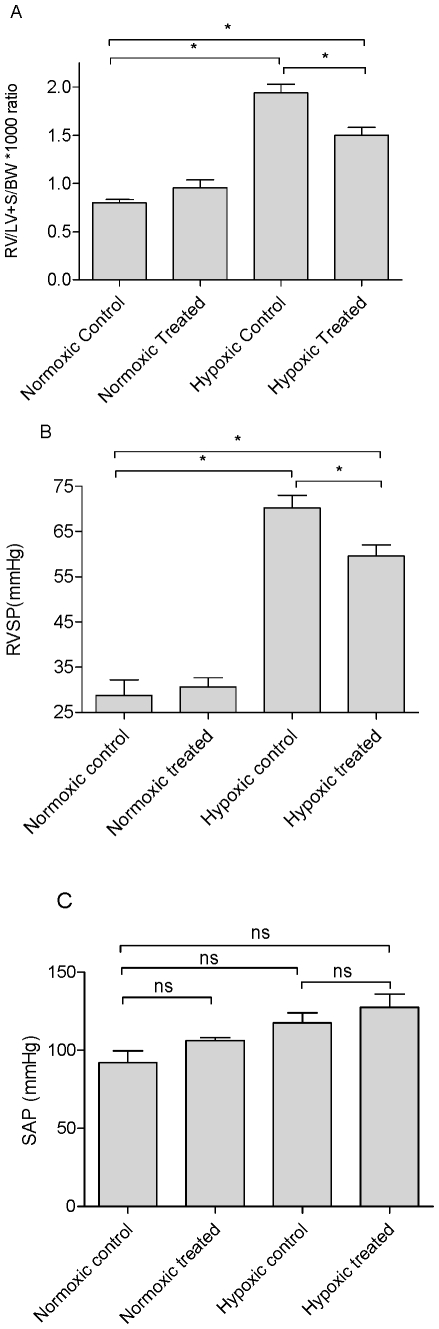
Effect of treatment with GW0742 on parameters of pulmonary hypertension in the chronic hypoxia rat model. Effect of normoxia versus hypoxia and treatment with GW0742 (30 mg/Kg) versus vehicle control male Sprague-Dawley rats on (A) the ratio of right ventricular to left ventricular plus septal weight and body weight, (B) the right ventricular systolic pressure (RVSP) and (C) the carotid systolic arterial pressure (SAP). The data is mean ± standard error of the mean for n = 4−6 for normoxic animals and 12–17 for hypoxic animals. Data were compared using one-way analysis of variance followed by a Bonferroni's multiple comparison test. Statistical significance was assumed where p<0.05 and is denoted by * and ns (not significant) where p<0.05.

**Figure 9:**
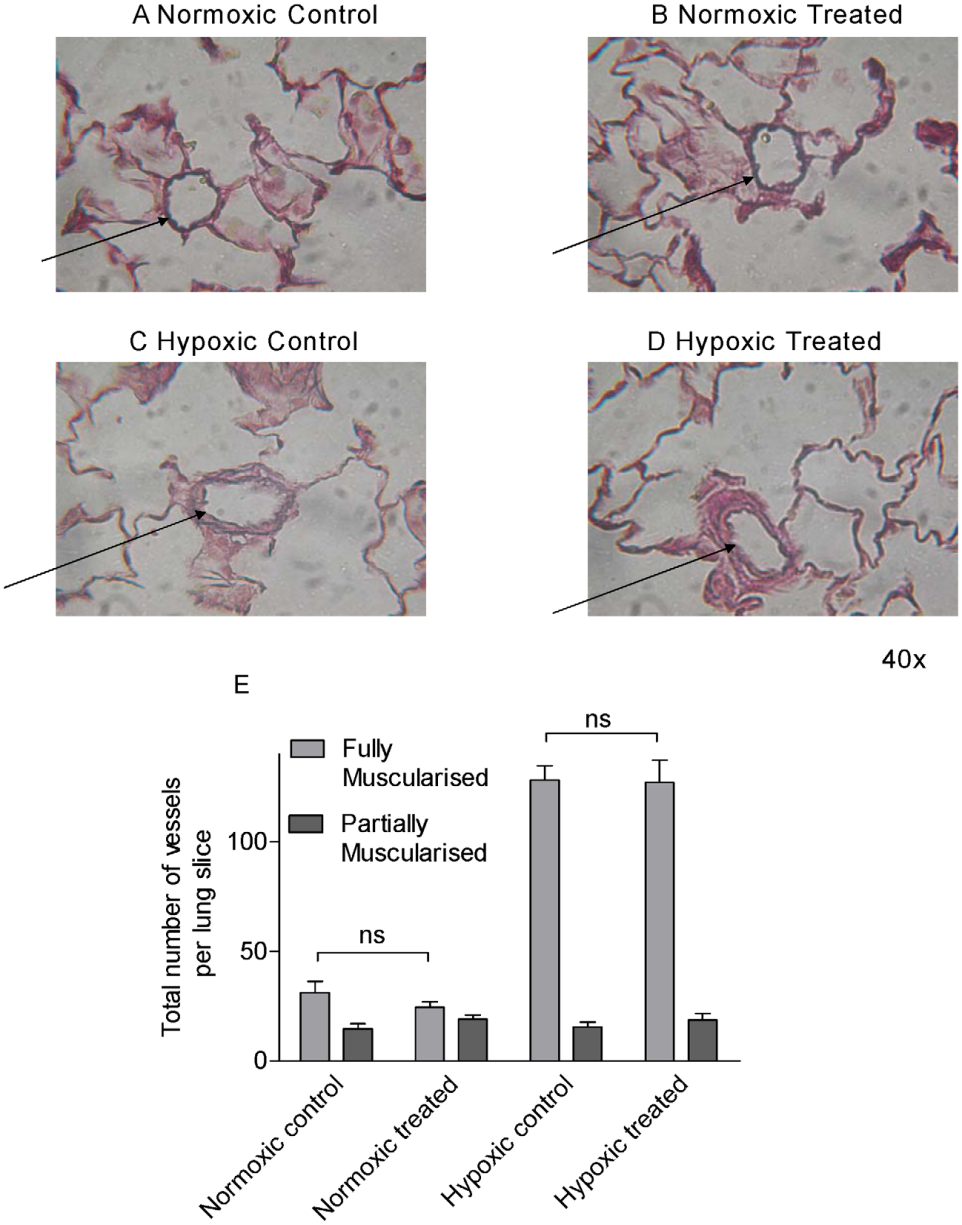
Effect of normoxia versus hypoxia and treatment with GW0742 (30 mg/kg) versus vehicle control on lung morphology and arteriole mascularisation in male Sprague-Dawley rats. Sections were stained with Van Giessen stain and pictures shown are representative samples. Histological features of remodelling secondary to hypoxia are the presence of a clear double elastic lamina and increased smooth muscle staining (indicated by arrows). Lungs were from animals in (A) normoxic, (B) normoxic treated with GW0742, (C) hypoxic or (D) hypoxic treated with GW0742 conditions. (E) Total number of fully muscularised compared with partially muscularised pulmonary arterioles in a single lung slice, mean ± standard error of the mean for n = 4−6 for normoxic animals and 12–17 for hypoxic animals.

Data from *in vitro* studies described above suggested that GW0742 may be therapeutically active in pulmonary hypertension. We therefore investigated the effects of GW0742 on physiological parameters in a rat model of hypoxia-induced pulmonary hypertension. Rats were exposed to hypoxia or air for 3 weeks and administered GW0742 (30 mg/kg oral administration daily) or vehicle. Drug treatments had no effect on body weight or haematocrit (Table 1). Hypoxia induced the cardinal signs of pulmonary hypertension including increased right heart mass (Figure 8a), increased right ventricular systolic pressure (Figure 8b) and remodelled pulmonary arteries (Figure 9). This study revealed significant reductions in both right ventricular hypertrophy (Figure 8a) and right ventricular systolic pressures (Figure 8b) in hypoxic animals treated with GW0742 compared to hypoxic controls. There were no significant differences in systolic arterial pressure between the groups (Figure 8c), although the trend appears to show higher systolic pressures in the GW0742-treated animals compared to controls. Finally, there were no significant differences seen in the vascular remodelling of distal pulmonary arterioles between the hypoxic control animals and hypoxic animals treated with GW0742 (Figure 9).

**Table 1:** Biometric data.

	Body weight at start of experiment (g+/−SD)	Body weight after 21 days hypoxia (g+/−SD)	Haematocrit (%+/−SD)
Normoxic control (n = 4)	237+/−6	295+/−22	42+/−3
Normoxic treated (n = 6)	236+/−10	292+/−17	44+/−3
Hypoxic control (n = 17)	263+/−16	285+/−22	64+/−8
Hypoxic treated (n = 12)	260+/−19	284+/−23	65+/−4

## Discussion

In the current study we have demonstrated that PPARβ/δ agonists induce relaxation of blood vessels, including pulmonary artery, and protect against right heart hypertrophy associated with hypoxia-induced pulmonary hypertension. These data suggest that PPARβ/δ agonists may have therapeutic utility in the treatment of pulmonary hypertension.

GW0742 relaxed three different murine blood vessels; the aorta, pulmonary artery and mesenteric artery. We also confirm our observations in mouse pulmonary artery by showing that GW0742 induces vascular relaxation in rat pulmonary artery. Interestingly, GW0742 was a more potent relaxant of pulmonary and mesenteric artery than it was of aorta. GW0742 relaxed pulmonary artery contracted with a range of stimuli including the thromboxane mimetic U46619, the adrenergic agonist phenylephrine and hypoxia applied *in vitro*. PPAR agonists as a class of drugs are currently being tested for their anti-inflammatory and therapeutic beneficial effects in a range of experimental models and clinical trials. Our data suggest that PPARβ/δ agonists may also be useful for the treatment of pulmonary hypertension. Similar suggestions have been made for the PPARγ agonist rosiglitazone [12]. However, we found that the two PPARβ/δ agonists we tested, GW0742 and GW501516, were more potent than the PPARγ agonist rosiglitazone as relaxing agents of pulmonary artery, although similar in potency as relaxants of aortic tissue. Bezafibrate was inactive as a relaxant of pulmonary artery and the weakest of the drugs as a relaxant of the aorta. This pharmacological analysis, whilst limited as it is based on *in vitro* protocols, suggests that PPARβ/δ agonists may be superior to PPARγ agonists in the treatment of pulmonary hypertension.

PPARs are classically thought of as regulators of gene induction via genomic and non genomic mechanisms. In the case of PPARβ/δ [25], as with other PPARs, the genomic pathway is thought to involve binding to retinoid X receptor (RXR) and the formation of heterodimers which then bind to response elements of target genes. PPARβ/δ also mediates gene induction via non genomic pathways linked to trans-repression of the anti-inflammatory protein BCL-6 [26]. Clearly the mechanism by which PPAR agonists dilate vessels acutely must be mediated independently of gene induction as the response is seen within minutes of adding the drug. Our group has shown that agonists of PPARβ/δ, including GW0742, inhibit platelet activation following just 5–10 minutes of treatment [11]. Clearly with such acute exposure, and as platelets have no nucleus, effects of PPARβ/δ agonists on platelet function must also be mediated independently of gene induction and the nucleus. Others have shown that agonists of PPARγ, such as rosiglitazone, have similar effects in platelets [27]. In platelets, our group has recently demonstrated that the non genomic inhibitory effects of PPAR agonists are associated with trans-repression of PKCα[28], [29]. However, inhibition of PKCα does not seem to explain fully the relaxant effects of PPARβ/δ agonists in blood vessels as, in our hands, the mixed PKC inhibitor Gö6983 did not mimic the effects of GW0742 in pulmonary artery. However, Gö6976, which also inhibits PKC, did induce limited relaxant responses (unpublished observations). How then do PPARβ/δ agonists relax blood vessels?

In blood vessels smooth muscle relaxation can be brought about by one or more well defined pathways [8]. These include the (i) nitric oxide-cGMP, (ii) adenylate cyclase-cAMP, (iii) RhoA kinase and (iv) activation of potassium channels leading to hyperpolarisation. In this study we show that the dilator effect of GW0742 in pulmonary artery was mediated independently of endothelial nitric oxide, as responses were not prevented by the nitric oxide synthase inhibitor L-NAME. We next explored the biochemical pathways that GW0742 might modulate in blood vessels in order to better understand the mechanism by which vessel relaxation occurs in response to this drug. Biochemical approaches are generally limited by tissue source and in these experiments we found that pulmonary artery was too small to obtain reliable samples after treatment and extraction. We therefore used aorta for biochemical studies because it is larger, providing more tissue for extraction, and can be cut into sections allowing for the inclusion of internal controls. We found that GW0742 did not increase cGMP or cAMP but did inhibit activation of the RhoA kinase pathway induced by U46619. Further, in order to investigate the effects of GW0742 on potassium channels we measured membrane potential in the smooth muscle component of mesenteric arteries incubated with GW0742. Mesenteric arteries were used for this protocol as the technique is well validated for this tissue. Results from these experiments were less clear. At concentrations where vasodilatation was approximately 75% of induced tone, no hyperpolarisation was detected. However, at maximal concentrations of drug we did note a significant hyperpolarisation response. Whilst it seems that these observations cannot explain completely the effects of GW0742 in the vasculature, they suggest a mechanism independent of cGMP and cAMP and implicate an action on RhoA kinase and a partial action on potassium channels. It should be noted however, that vessels of different anatomical locations can utilise different signalling pathways. The mechanism by which GW0742 induces vascular relaxation in the pulmonary circulation remains the subject of investigation.

GW0742 is a potent activator of PPARβ/δ receptors with EC_50_ concentrations in the low nM range [30]. Vascular relaxation induced by GW0742 of vessels was seen in the µM range. It is therefore possible that ‘PPARβ/δ agonists’ are inducing effects on blood vessels independent of the target receptor. In order to address this we compared responses of GW0742 in blood vessels from wild type and PPARβ/δ^−/−^ mice. Interestingly the relaxant effects of GW0742 on pulmonary or mesenteric artery appeared to be independent of the target receptor PPARβ/δ. When responses were analysed in aorta however, there was a significant blunting in the relaxant responses induced by GW0742. In a recent study from our group on platelet responses of GW0742, we also found that some, but not all inhibitory effects of GW0742 were mediated by PPAR β/δ [31]. These observations are surprising, but interesting and show that off-target effects of drugs designed to activate PPARβ/δ may have additional beneficial effects.

Prostacyclin is an endogenous hormone ligand for PPARβ/δ receptors. However, prostacyclin also activates the cell surface G-protein coupled IP receptor. We reasoned that molecules could display promiscuity between these two types of receptors and so investigated the role of IP in dilator effects of GW0742. GW0742 induced comparable vascular relaxant effects in mesenteric arteries from IP^−/−^ mice as seen in tissue from wild type animals. However, there was a small, but statistically significant blunting of the ability of GW0742 to dilate pulmonary artery from IP^−/−^ mice compared to tissue from wild type animals. This data suggests that whilst IP receptors may play a small role in the effects of GW0742 in some vessels, the major functional effect of this drug is, on the whole, independent of IP receptors.

All of the above evidence suggests that GW0742 is a good drug candidate for the treatment of pulmonary hypertension. We therefore investigated this possibility directly by studying the effects of GW0742 on pathophysiological symptoms of hypoxia-induced pulmonary hypertension in the rat. Rats placed in hypoxic chambers for three weeks developed cardinal signs of pulmonary hypertension including increased right ventricular systolic pressure, increased right heart mass and dramatic remodelling. In this study, as in others [22], [23], right ventricular systolic pressure was used as a surrogate for mean pulmonary artery pressure. However, it is acknowledged that this parameter can be influenced by cardiac effects, independent of pulmonary vascular responses. Animals were treated with GW0742 or vehicle by daily oral dosing for the entire duration of the model. GW0742 had no significant effects on physiological parameters measured in rats kept under normoxic conditions. Despite the findings that GW0742 relaxes a range of blood vessels, it was interesting that rats treated with GW0742 had no significant reduction in systemic arterial pressure. This is also true for other pulmonary hypertension drugs which relax blood vessels such as sildenafil and prostacyclin (or mimetics such as trepostinil sodium etc). In the case of sildenafil and prostacyclin systemic effects can be seen in patients if doses are elevated, but a therapeutic window clearly exists for these drugs outside systemic pressure effects [32]. In the case of GW0742 and our study, it may be relevant that the relaxant effects of the drug were more potent on pulmonary and mesenteric vessels than on the aorta – it is possible that over all GW0742 dilates selected beds (including the pulmonary circulation). However, it is equally possible that the effective concentration of GW0742 *in vivo*, like those achievable with sildenafil and trepostinil sodium, fall below that required to affect systolic pressures.

Hypoxic animals treated with GW0742 displayed significant reductions in right ventricular systolic pressure and in right heart mass but, Interestingly, GW0742 had no effect on the remodelling seen in arterioles of hypoxic animals. Changes in right ventricular systolic pressure and right heart mass, without changes in arteriole remodelling, suggest GW0742 is affecting pulmonary vascular tone directly and are very much in line with what we have seen of this drug on tissue vessels *in vitro*. However, as discussed above, high concentrations of GW0742 are required to induce vessel relaxation. It should therefore also be considered that the effects of GW0742 in the *in vivo* model may result from an action directly on the right ventricle. This possibility remains the subject of investigation.

In the clinic, patients with pulmonary hypertension are currently treated with oral phosphodiesterase type V inhibitors (such as sildenafil) and/or ET receptor antagonists (such as bosentan). As outlined above, GW0742 induces vasodilatation independently of either of these pathways. Moreover, in animal models like the one used here in our study sildenafil [24] or bosentan [33] reduce arteriole remodelling. Thus, considering the clinical profile and signalling mechanism involved, we suggest that GW0742 will be an effective co-treatment with either phosphodiesterase type V or ET-1 receptor antagonists for patients with pulmonary hypertension.
